# Human Embryonic Stem Cells Derived from Embryos at Different Stages of Development Share Similar Transcription Profiles

**DOI:** 10.1371/journal.pone.0026570

**Published:** 2011-10-21

**Authors:** Gnanaratnam Giritharan, Dusko Ilic, Matthew Gormley, Ana Krtolica

**Affiliations:** 1 SLL Sciences, StemLifeLine, Inc., San Carlos, California, United States of America; 2 University of California San Francisco, San Francisco, California, United States of America; Baylor College of Medicine, United States of America

## Abstract

We have derived hESC from biopsied blastomeres of cleavage stage embryos under virtually the same conditions we used for the derivation of hESC lines from inner cell mass of blastocyst stage embryos. Blastomere-derived hESC lines exhibited all the standard characteristics of hESC including undifferentiated proliferation, genomic stability, expression of pluripotency markers and the ability to differentiate into the cells of all three germ layers both *in vitro* and *in vivo*. To examine whether hESC lines derived from two developmental stages of the embryo differ in gene expression, we have subjected three blastomere-derived hESC lines and two ICM-derived hESC lines grown under identical culture conditions to transcriptome analysis using gene expression arrays. Unlike previously reported comparisons of hESC lines which demonstrated, apart from core hESC-associated pluripotency signature, significant variations in gene expression profiles of different lines, our data show that hESC lines derived and grown under well-controlled defined culture conditions adopt nearly identical gene expression profiles. Moreover, blastomere-derived and ICM-derived hESC exhibited very similar transcriptional profiles independent of the developmental stage of the embryo from which they originated. Furthermore, this profile was evident in very early passages of the cells and did not appear to be affected by extensive passaging. These results suggest that during derivation process cells which give rise to hESC acquire virtually identical stable phenotype and are not affected by the developmental stage of the starting cell population.

## Introduction

Totipotency in human embryos persists until 4–8 cell stage [1], [2]. Subsequently, genome activation initiates differentiation, with certain blastomeres forming the outer, polar, trophectoderm while others retain their pluripotent potential and generate the non-polar inner cell mass that will give rise to the future organism. These two morphologically distinct cell populations continue to divide as they form the blastocyst, and can easily be physically separated. Human embryonic stem cells (hESC) are typically derived from the pluripotent inner cell mass cells of the blastocyst [3]. More recently, we and others have reported alternative method of hESC derivation from biopsied blastomeres isolated from 8-cell/cleavage stage embryos [4], [5]. While blastomeres of the 8-cell stage embryo and the cells of the ICM show some biological similarities, including a non-polarized phenotype and pluripotent potential, they also exhibit significant differences. Indeed, microarray analysis of gene expression during six developmental stages of human preimplantation embryos showed a group of 2299 probe sets differentially expressed in blastocyst stage compared to 8-cell stage embryos [6]. The majority of these genes were involved in lipid and fatty acid metabolism. In addition, a group of 1715 probe sets involved in the regulation of transcription and nucleic acid metabolism were down-regulated in blastocysts compared to 8 cell stage embryos [6]. Furthermore, the DNA of the blastomeres of the 8 cell stage embryos is completely demethylated as a result of reprogramming and activation of embryonic genome whereas the methylation marks are already re-established in the cells of the ICM [7]. In addition, blastomeres of the 8-cell stage embryo exhibit lower telomerase activity and shorter average telomere length than cells in the ICM [8].

During the derivation process, hESC acquire a polarized phenotype, a characteristic not present in the pluripotent mouse ESC [9]. Indeed, they appear closer to mouse stem cells derived from primitive ectoderm than those from the inner cell mass. Similarly, the active molecular signaling pathways in hESC resemble those of mouse epiblast stem cells derived from post-implantation embryo [10]. This indicates that hESC derived from the ICM acquire characteristics typical of more advanced stages of development. Hence, we investigated whether human stem cell lines derived from late totipotent blastomeres of the cleavage stage embryo develop a more “primitive” phenotype than ICM-derived lines by profiling their respective gene expression, molecular regulation and signaling pathways. In this study, we examined whether the inherent differences in the developmental stage of the starting embryonic cell population are maintained in the derived hESC lines by evaluating the stemness, differentiation capacity and gene expression profiles of blastomere- and ICM-derived hESC lines.

## Materials and Methods

### Ethics Statement

Derivation of hESC lines has been conducted in accordance with the study protocol approved by Western Institutional Review Board (WIRB).

### Derivation and culture of hESC

**Figure 1:**
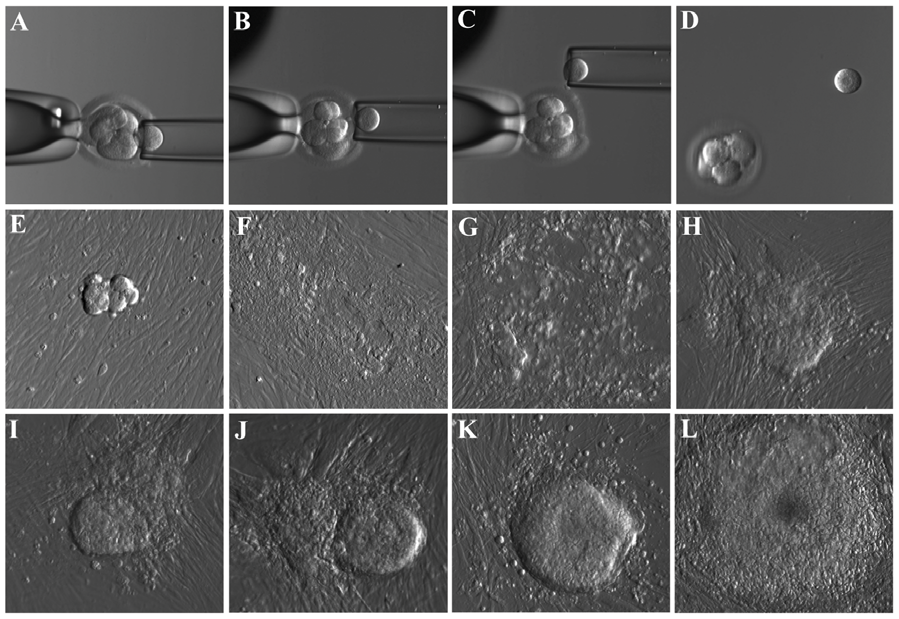
Derivation of hESC from biopsied blastomere of the cleavage stage embryo. Micrographs showing the stepwise procedure of embryo biopsy using inverted microscope-attached micromanipulator (A–D) and the appearance of initial outgrowth and hESC colony during the derivation procedure (E–L). All pictures were taken using microscope mounted digital camera and Hoffman optics under 200X total magnification.

Three human embryonic stem cell (hESC) lines were derived from biopsied blastomeres of 2 surplus frozen human cleavage stage embryos donated by two consenting couples that provided written consent according to the protocol approved by WIRB. These lines (W10, SAB-113B, and SAB-113D) were derived using established derivation procedure in our laboratory (illustrated in Fig. 1). Briefly, the embryos were thawed and incubated in Quinn's cleavage medium for minimum 3 h at standard culture conditions. Blastomeres were removed from each embryo using established biopsy procedure [11]. Each biopsied blastomere was cultured in a 20 µl drop of Quinn's cleavage medium in an incubator at 37 °C. After 24 h incubation, the blastomeres were transferred onto irradiated human foreskin fibroblast (HFF) feeders in 50 µl drops of Quinn's cleavage medium supplemented with human laminin (10 µg/ml; day 0). Three days later cultures were assessed for blastomere attachment to feeders. Starting on day 3, medium in drops containing attached blastomeres has been refreshed every day by replacing 1/3 of volume with Quinn's blastocyst medium supplemented with human laminin (10 µg/ml), recombinant human leukemia inhibitory factor (LIF; 10 ng/ml), and recombinant human basic fibroblast growth factor (FGF; 25 ng/ml; R&D Systems, Inc.). From day 5, Quinn's blastocyst medium was replaced with standard hESC medium (80% KO-DMEM, 20% KSR, FGF-25 ng/ml, and LIF-10 ng/ml), and replaced in drops daily. Embryonic stem cell-certified fetal calf serum (10%) was added to the medium for a week. On day 11, initial hESC colonies were dissected and left in the same drop. On day 14, colonies were dissected and transferred into 4-well dish with new HFF feeders. From the next day onwards the cells were cultured in standard hESC medium containing FGF (25 ng/ml) and LIF (10 ng/ml).

### Immunocytochemistry and alkaline phosphatase assay

The expression of pluripotency markers such as OCT4, NANOG, SSEA4, TRA-1-60 and TRA-1-81, and the differentiation markers of all 3 germ layers such as β-III tubulin, smooth muscle actin and α-feto protein, were evaluated by immunocytochemical analysis. Briefly, hESC colonies and differentiated cells were fixed in 4% paraformaldehyde (PFA) or 90% acetone at room temperature for 10 min. The PFA-fixed cells were permeabilized in 0.1% Triton at room temperature for 10 min. The cells were then, incubated with primary antibodies overnight at 4 °C, and stained with fluorescent dye-conjugated donkey secondary antibodies. The immunostained cells were mounted in Vectashield (Vector Laboratories, Inc., Burlingame, CA, USA) and evaluated under fluorescent microscope (Nikon Eclipse 50i, Nikon Instruments Inc., Melville, NY, USA). To evaluate the expression and activity of alkaline phosphatase in the undifferentiated hESC, ELF Phosphatase Detection Kit (ATCC, Manassas, VA, USA) was used and the kit manufacturer's instructions were followed. Briefly, the cells were fixed in 4% PFA at room temperature for 10 min and permeabilized in 0.2% Tween-20 solution at room temperature for 10 min. Next, the substrate was added and incubated at room temperature. After 5 min incubation, the reaction was stopped and cells were washed 3 times using wash buffer. Cells were mounted and observed under fluorescent microscope.

### Flow cytometry

Flow cytometry was performed using PerCP-Cy5.5 conjugated anti-human OCT4 antibody and PE conjugated anti-human NANOG antibody (BD Biosciences, San Jose, CA, USA) and fixation and permeabilization reagents A and B (Invitrogen Corp., Carlsbad, CA, USA). hESC colonies were accutase treated (to separate into single cells) and fixed in reagent A at room temperature for 15 min. Next, cells were permeabilized in reagent B at room temperature for 5 min and incubated with antibodies at 4 °C for 20 min. The cells were resuspended in sheath fluid and flow cytometry was performed using a flow cytometer (BD LSR II, BD Biosciences).

### *In vitro* and *in vivo* differentiation of hESC

For *in vitro* differentiation, colonies were cultured on feeders in standard hESC medium containing 10% fetal bovine serum without FGF. After 2–3 week differentiation, cells were fixed in 4% paraformaldehyde (PFA) for immunocytochemistry. Cells were also collected for RNA extraction and RT-PCR was performed to evaluate the expression of differentiation markers. For *in vivo* differentiation, 5–6 million hESC were mixed with 30% matrigel and transferred on ice to Jackson West animal facility (The Jackson Laboratory – West, Sacramento, CA, USA) where they were subcutaneously injected under the dorsal flank of immunocompromised NOD/SCID mice. The animals were observed for teratoma formation and sacrificed 3 months after injection. Histopathological analysis was performed on the sections of the teratoma to verify the formation of tissues from all three germ layers and lack of invasive tumors.

### RNA extraction and RT-PCR

RNA was isolated using RNAeasy Plus Minikit (Qiagen Inc., Valencia, CA, USA) and converted to cDNA using SuperScript III CellsDirect cDNA Synthesis System (Invitrogen). The polymerase chain reaction (PCR) was performed using Platinum PCR SuperMix (Invitrogen) and primers for transcripts of markers from all three germ layers: *T* (Brachyury protein), *WT1* (Wilms tumor) and *ACTA2* (smooth muscle actin, α2) were chosen as markers of mesoderm, *GATA4* (GATA binding protein 4), *SOX17* (sex determining region Y – box 17) and *AFP* (α fetoprotein) as markers of endoderm, and *TUBB3* (Tubulin, β3), *NES* (nestin) and *PAX6* (paired box 6) as markers of ectoderm. The PCR products were analyzed by gel electrophoresis using ethidium bromide (1 µg/mL) stained 2% agarose gels and photographed under ultraviolet illumination. The primer sequence, annealing temperature, number of PCR cycles and fragment size are provided in Table S1.

### Karyotype analysis

Karyotype analysis (G banding) of each hESC line has been performed at certified Cytogenetics Laboratory, Children's Hospital and Research Center, Oakland, CA, USA on early and later passages of each hESC line.

### Cell culture and sample collection

Samples from 3 of the blastomere derived hESC lines (SAB-113B, SAB-113D and W10), and 2 of the whole embryo derived hESC lines (SG4 and SG7) established in our laboratory were collected for microarray analysis. All the selected hESC lines were adjusted to grow under feeder-free defined culture conditions on defined human matrix (CellStart, Invitrogen)-coated dishes in StemPro SFM serum-free hESC medium (Invitrogen) containing FGF (10 ng/ml), and passaged using accutase (Millipore, Temecula, CA, USA) for enzymatic cell separation. For each hESC line, cells were collected when the colonies showed 70–80% confluency in three biological replicates for SAB-113B and SAB-113D lines and 2 replicates for W10, SG4 and SG7 lines. Cells were washed once in DPBS, pelleted, snap frozen in liquid nitrogen and stored at −80 °C. Frozen cell pellet samples and human universal reference total RNA from 2 different lot numbers (Catalog number 636538, Clontech, Mountain View, CA, USA) were sent to Expression Analysis (Expression Analysis, Inc., Durham, NC, USA) to conduct microarray analysis using Human Genome U133 Plus 2.0 gene chips (Affymetrix, Santa Clara, CA, USA).

### Microarray data analysis

Statistical analysis was carried out with Affymetrix CEL files using the software environment R (http://www.r-project.org/) and the Bioconductor packages implemented within affylmGUI [12], [13]. In order to remove possible sources of variation of a non-biological origin between arrays, intensity values between arrays were normalised using the robust multiarray averaging (RMA) and log2 expression values exported [14]. The top 10,000 probe sets (based on summed median difference from reference RNA) were subject to unsupervised hierarchical clustering (with sample/probe leaf ordering optimized, Pearson correlation, average link clustering) using MeV (http://www.tigr.org/) [15]. The top 25 and bottom 25 genes are presented. Differential expression of genes between treatment groups was determined by linear modelling and empirical Bayes statistics. Differentially expressed genes were selected using a fold-change cut-off value of 2 and above, BH corrected p score of less than 0.01, and a B score of more than 5. Functional annotations were carried out using Affymetrix annotation data to identify overrepresented gene ontology functions of uniquely expressed genes in blastomere and whole embryo derived hESC lines compared to reference RNA. Ingenuity Pathways Analysis platform (http://www.ingenuity.com/) was used to identify the statistically significant genes involved in the pathways of stemness regulated by *OCT4* and *NANOG*, in lines of both origins compared to reference RNA.

## Results

### Derivation and characterization of hESC lines from biopsied blastomeres of the cleavage stage embryo

**Figure 2:**
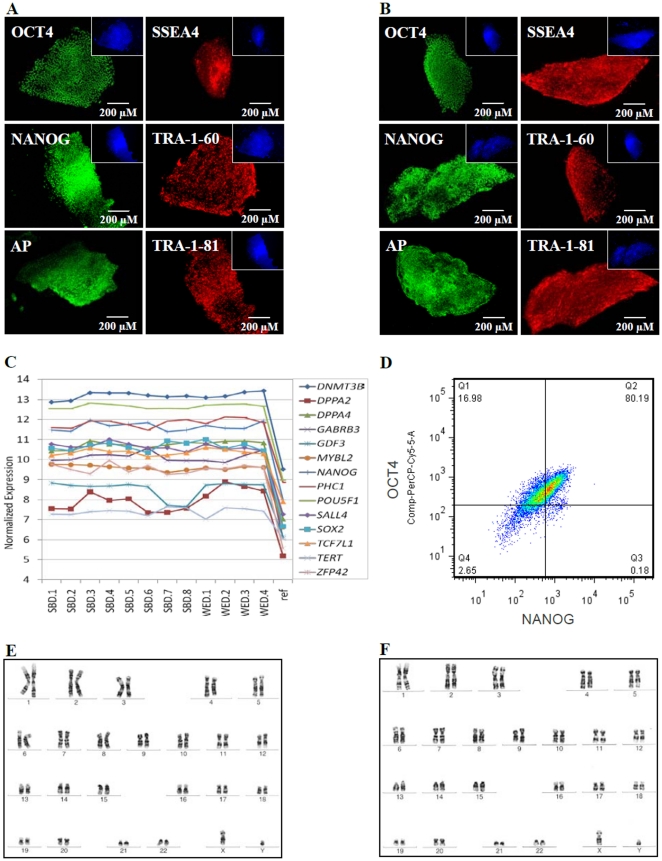
Expression of pluripotency genes in cleavage stage blastomere-derived and blastocyst stage ICM-derived hESC. SAB-113B (A) and SAB-113D (B) cells express markers of pluripotency as shown by immunocytochemical analyses (OCT4, NANOG, SSEA-4, TRA 1-60, and TRA 1-81) and alkaline phosphatase activity (AP). Insets of the images show the nuclei of the cells stained with HOECHST 33342. Normalized expression of well-known 14 pluripotency genes (*DNMT3B, DPPA2, DPPA4, GABRB3, GDF3, MYBL2, NANOG, PHC1, POU5F1, SALL4, SOX2, TCF7L1, TERT* and *ZFP42*) in all the replicates of blastomere lines (SBD1, SBD2, SBD3, SBD4, SBD5, SBD6, SBD7 and SBD8), and whole embryo lines (WED1, WED2, WED3 and WED4) showed consistent high values compared to reference RNA (ref) (C). Flow cytometric analysis of these lines also showed consistent high expression of OCT4 and NANOG. Representative diagram for SAB-113B is depicted (D). In addition, SAB-113B (E) and SAB-113D (F) lines exhibited a stable male karyotype (46, XY) after 10 passages in culture.

Using previously described methods for blastomere biopsy and hESC derivation on human feeders, we have derived two new hESC lines from biopsied blastomeres of cleavage (8-cell) stage embryos (Fig. 1). These newly derived hESC lines, SAB-113B and SAB-113D, expressed known markers of stemness including OCT4, NANOG, SSEA4, TRA-1-60, TRA-1-81 as determined by immunocytochemical analysis and exhibited alkaline phosphatase activity (Fig. 2A, 2B). Similar results have already been obtained for previously derived blastomere line W10 [4] and whole embryo lines SG4 and SG7 (data not shown). Next, we compared normalized expression of 14 established hESC stemness genes (*DNMT3B, DPPA2, DPPA4, GABRB3, GDF3, MYBL2, NANOG, PHC1, POU5F1, SALL4, SOX2, TCF7L1, TERT* and *ZFP42*) in newly generated blastomere-derived SAB-113B and SAB-113D lines, previously derived W10 blastomere line, and whole embryo ICM-derived lines, SG4 and SG7, and observed consistent high expression of all stemness genes examined and lack of any major differences in their expression levels between blastomere-derived and whole embryo-derived hESC lines (Fig. 2C). Furthermore, FACS analysis showed that more than 80% of cells in blastomere-derived (Fig. 2D) and whole embryo ICM-derived (data not shown) hESC lines expressed both OCT4 and NANOG. In addition, these lines also showed no significant difference in growth rate, with approximate doubling time of 40 h on feeders and 24 h under feeder-free conditions. Cell and colony morphology was examined semiweekly by inverted microscopy using phase contrast light and Hoffman optics and did not reveal any significant differences between these lines. Newly derived hESC lines exhibited stable normal male karyotype (46, XY) after 10 passages in culture (Fig. 2E, 2F) as examined by G-banding analysis. Blastomere-derived W10 line showed a stable female karyotype (46, XX) as previously reported and both whole embryo SG4 and SG7 lines exhibited a stable normal male karyotype (46, XY; data not shown).

### *In vitro* and *in vivo* differentiation

**Figure 3:**
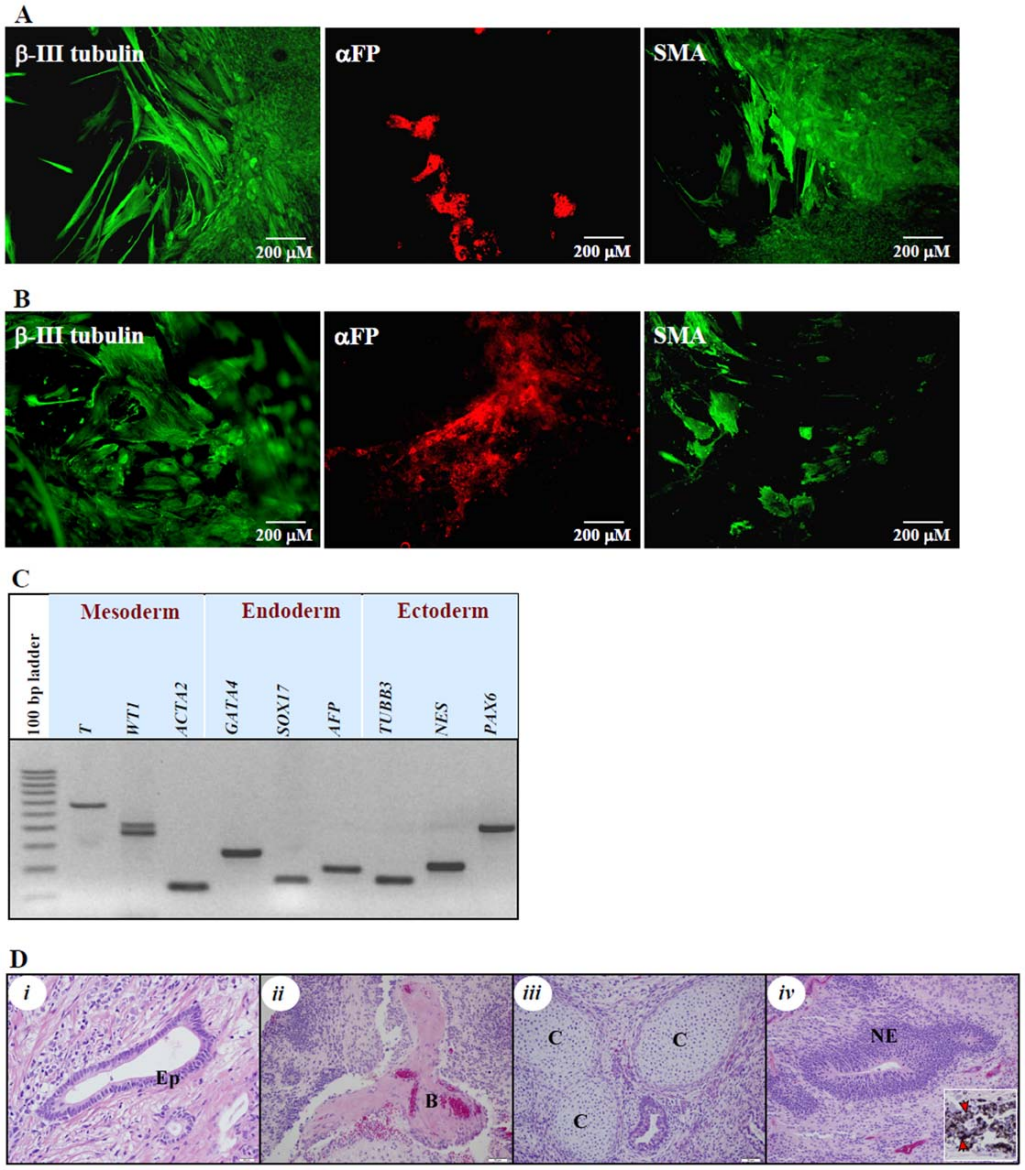
Blastomere-derived hESC lines, SAB-113B and SAB-113D, can be differentiated into derivatives of all three germ layers. *In vitro* differentiation of SAB-113B (A) and SAB-113D (B) into all 3 germ layers was confirmed by immunocytochemical analysis. αFP – α fetoprotein (endoderm), β3T – βIII-tubulin (ectoderm), SMA – smooth muscle actin (mesoderm). The expression of derivatives of mesodermal – T (Brachyury homolog), WT1 (Wilms tumor 1) and ACTA2 (smooth muscle actin, α2); endodermal – GATA4 (GATA binding protein 4), SOX17 (sex determining region Y- box 17) and AFP (α-fetoprotein); and ectodermal – TUBB3 (tubulin, β3), NES (nestin) and PAX6 (paired box 6) genes were also confirmed by RT-PCR analysis (C). *In vivo* differentiation was confirmed by histopathological analysis of teratoma tissue obtained 3 months post-initiation under the dorsal flank of immunocompromised NOD/ SCID mice (D). Images of representative areas of H & E stained histological sections of the tumors: derivatives of endoderm – epithelial tissue (Ep) (*i);* mesoderm – bone tissue fragment (B) *(ii)* and early cartilage tissue (C) *(iii)*; and ectoderm – neuroectodermal tissue (NE) *(iv)* and cells with melanin granules (red arrowheads) (*iv*, inset).

hESC derived from the ICM of blastocyst stage embryos have the inherent capacity to differentiated into tissues of all three germ layers both in culture and when injected into the animals. To examine whether our newly derived blastomere lines exhibit similar pluripotent capacity, we have subjected SAB-113B and SAB-113D lines to spontaneous *in vitro* differentiation and teratoma formation *in vivo*. Differentiation in culture was performed in serum-containing differentiation medium on human matrix-coated plates. After 2–3 weeks of differentiation, cells were examined by RT-PCR and immunocytochemical analysis to determine expression of markers of all 3 germ layers. Our data showed that upon differentiation, similarly to ICM-derived lines, blastomere-derived lines also expressed markers of all three germ layers (Fig. 3A–C). We have previously reported that the differentiated products from blastomere-derived W10 line also showed similar expression of markers of all 3 germ layers.

To assess *in vivo* differentiation capacity of blastomere-derived lines, 5–6 million cells were subcutaneously injected under the dorsal flank of immunocompromised NOD/ SCID mice and tumors were extracted when they reached 100 mm^3^. The histopathological analyses of the teratomas revealed that the hESC derived from blastomeres form benign tumors and differentiate into tissues of all three germ layers (Fig. 3D).

### Transcriptome analysis

**Figure 4:**
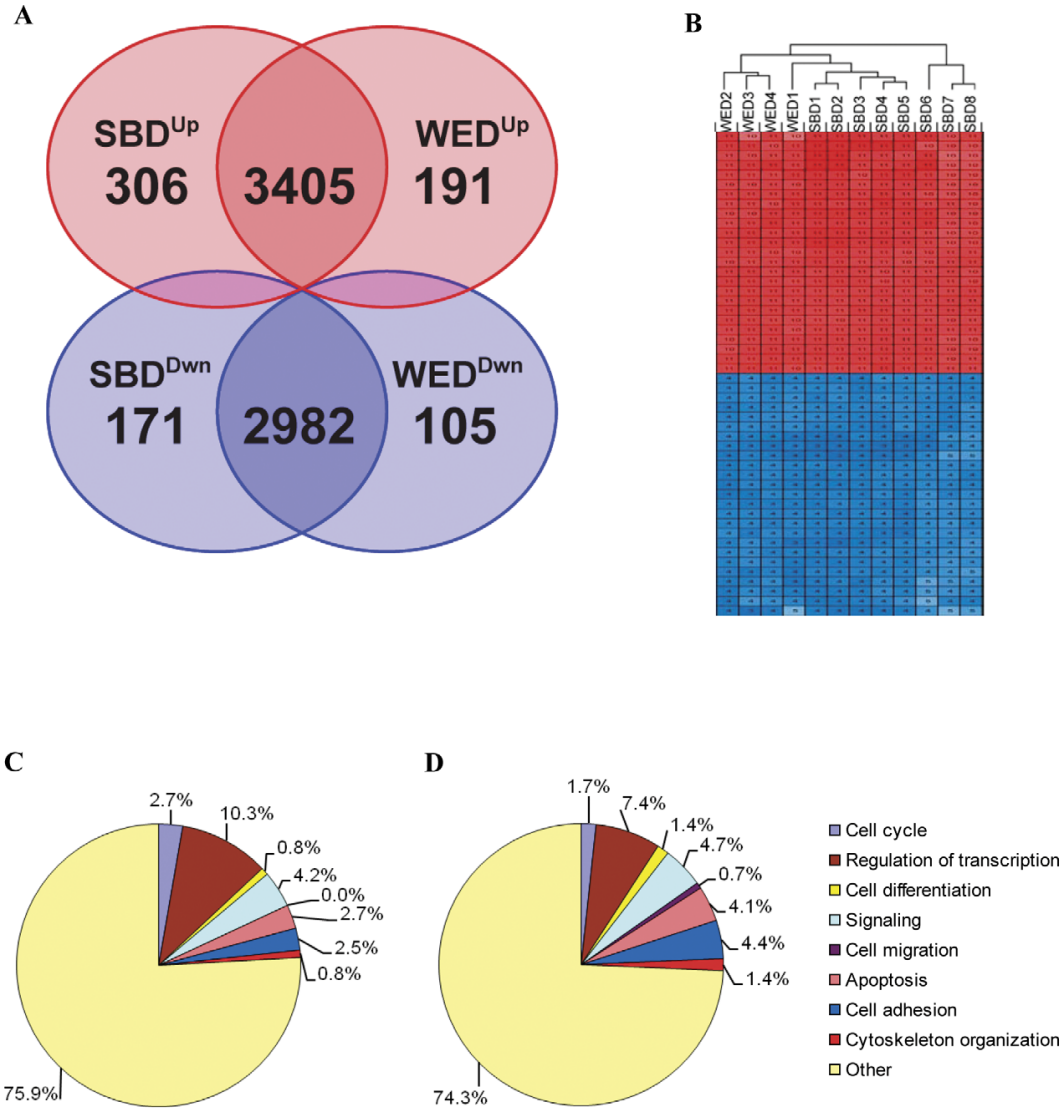
Gene expression analysis of cleavage stage blastomere-derived and blastocyst stage ICM-derived hESC lines. Venn diagram of microarray analysis showing differentially expressed genes after pairwise comparison of blastomere-derived (SBD) and whole embryo-derived (WED) lines to reference RNA (A). Clustering tree generated by unsupervised hierarchical clustering analysis of summed median difference in the top 10,000 probe sets relative to reference RNA, and the normalized expression value of top 25 overexpressed and top 25 underexpressed genes (B). Blue and red colors represent lower and higher expression, respectively. Pie charts depict distribution of uniquely expressed genes of blastomere-derived (C) and whole embryo-derived (D) lines compared to reference RNA into known cellular biological processes such as cell cycle, regulation of transcription, cell differentiation, signaling, cell migration, apoptosis, cell adhesion, and cytoskeleton organization.

We performed transcriptome analysis on two whole embryo-derived lines (WED) from blastocyst-stage (SG4 and SG7) and three single blastomere derived lines (SBD) from cleavage stage embryos (SAB-113B, SAB-113D and W10). SAB-113B and SAB-113D lines were derived from two different blastomeres of one embryo and W10 line was derived from a single blastomere of another embryo. The SG4 and SG7 lines were derived from ICM of two different whole embryos. Three replicates of SAB-113B, SAB-113D blastomere lines, and two replicates of W10 blastomere line and SG4 and SG7 whole embryo lines, and human universal reference total RNA from two different lot numbers were included in the experiment. In order to minimize culture induced variations in gene expression, all blastomere and whole embryo lines were grown under identical defined feeder-free serum-free culturing conditions on human matrix-coated surfaces. Cell pellets were collected and RNA samples were generated from cells harvested when the colonies reached 70–80% confluency to ensure similar cell cycle profiles. Human Universal Reference Total RNA was used as control to identify genes which are specifically over- and under- expressed in hESC lines. These commercially available RNA samples were made by pooling total RNA extracts from a collection of different whole tissue sources, thus providing the broadest coverage of expressed genes. The similarities and differences between the single embryo derived (SBD) and whole embryo derived (WED) lines were established by first comparing each to the reference RNA, then performing a 4-way venn of the identified up- and down-regulated genes. Of the 6894 and 6683 significantly different genes in SBD and WED respectively (relative to reference RNA), some 6387 were shared (over 90%). Specifically, 3405 genes were commonly up-regulated and 2982 were commonly down-regulated. None of the genes were oppositely regulated (Fig. 4A). This indicates that less than 10% of the genes may be uniquely expressed in ESC line of specific origin.

Based on summed median difference of normalized expression value of samples from reference RNA, the top 10,000 probe sets were subject to unsupervised hierarchical clustering, with the normalized expression values of top 25 overexpressed and top 25 underexpressed genes and sample dendrogram represented in Figure 4B. Hierarchical clustering revealed that the SBD and WED lines do not show a clear separation, indicating that hESC lines grown under identical conditions share similar gene expression profiles, independent of the developmental stage of the embryo from which lines originate (Fig. 4B).

In both blastomere- and whole embryo-derived ESC lines, only about one-quarter of the unique genes, whose expression differs from reference RNA, were associated with an annotated biological processes such as cell cycle, regulation of transcription, cell differentiation, signaling, cell migration, apoptosis, cell adhesion and cytoskeleton organization (as categorized by GO, as listed in the Affymetrix gene annotation data). Thus, the potential role of the majority of altered genes controlling the establishment and maintenance of hESC phenotype remains to be elucidated (Fig. 4C, 4D). It is quite likely that some of the expressed transcripts do not have a functional role, are not translated and could be by-product of the open chromatin structure. Among GO annotated genes, signaling, apoptosis and cell adhesion showed about 0.5 to 1.9% less unique genes in the blastomere-derived lines compared to whole embryo-derived lines. While 0.7% of unique genes from whole embryo lines were associated with cell migration, blastomere derived lines did not have altered expression of any of genes from this category. In blastomere derived lines, there was 1% increase in unique genes associated with cell cycle and 2.9% increase in genes involved in regulation of transcription.

**Figure 5:**
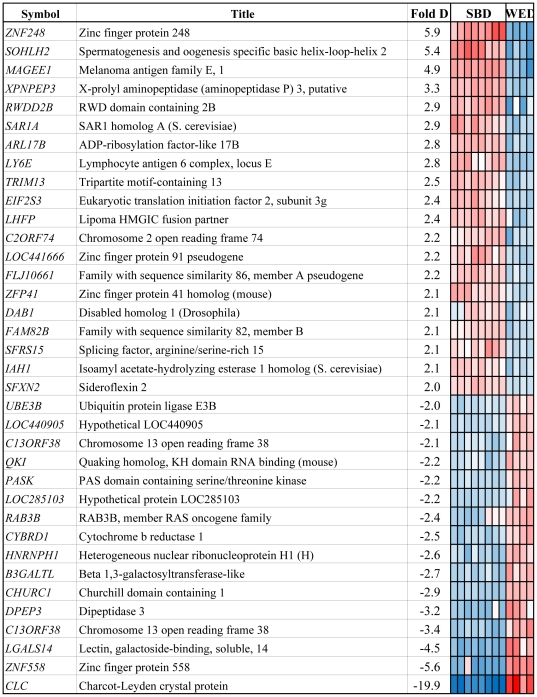
Differentially expressed genes with fold change after direct comparison of cleavage stage blastomere-derived (SBD) and blastocyst stage whole embryo-derived (WED) lines. Blue and red colors represent lower and higher expression, respectively.

To identify the genes which show significant differences between blastomere and ICM-derived lines, direct pair-wise comparison was performed. The analysis revealed only 36 annotated genes (20 genes upregulated in blastomere derived lines relative to ICM-derived and 16 genes downregulated) were significantly different between the lines of both origins (Fig. 5). Out of these 36 genes, only 6 genes (3 upregulated - *ZNF248*, *SOHLH2* and *MAGEE1,* and 3 downregulated - *CLC*, *ZNF558* and *LGALS14*) showed more than 4 fold changes in expression between these hESC lines indicating that only minor differences exist. These include three DNA binding proteins with potential role in regulation of gene transcription: ZNF248, ZNF558 and SOHLH2 and proteins with potential membrane functions: MAGEE1, LGALS14 and CLC (Charcot-Leyden crystal protein/Galectn-10). *ZNF248* is a Krueppel C2H2-type zinc-finger DNA binding protein with potential role in transcriptional regulation [16]. *ZNF558* is zinc finger DNA binding protein that also binds to Rrp46 which is an exosome subunit and mRNA splicing/processing factor with potential role in mRNA degradation and gene expression [17]. *SOHLH2* is a spermatogenesis- and oogenesis-specific basic helix–loop–helix (bHLH) transcription factor implicated in regulation of early germ cell development [18]. *MAGEE1* gene is an X chromosome gene and encodes an α-dystrobrevin-associated MAGE (melanoma-associated antigen) protein (DAMAGE) that may have a signaling role in brain, muscle, and peripheral nerve [19]. *LGALS14* gene encodes a galectin family protein predominantly expressed in placenta [20]. *CLC* gene encodes lysophospholipase, enzymes that regulate the lysophospholipids in biological membranes [21]. It is related to the galectin family and may also possess carbohydrate or IgE-binding activities, and be associated with inflammation and some myeloid leukemias [22].

A number of the genes, which showed 2–4 fold difference, do not have known function. Other genes, that are either upregulated *(SFRS15, DAB1, LHFP, XPNPEP3, LY6E, SAR1A, EIF2S3 and TRIM13*) or downregulated (*UBE3B, QK1, PASK, RAB3B*) in blastomere- relative to the whole embryo-derived lines, have been assigned specific functions. For example, *SFRS15* product binds to carboxyl-terminal domain of the Rpb1 subunit of RNA polymerase II and regulates the transcription [23], [24]. *DAB1* encodes a scaffold protein which interacts with cytoplasmic tails of certain low density lipoprotein (LDL) receptor family proteins that regulate tyrosine kinase signaling and the remodeling of cytoskeleton [25]. *LHFP* acts as a partner in HMGIC translocations in lipoma [26]. *XPNPEP3* encodes a prolyl aminopeptidase which is localized to mitochondria and also has ciliary function in renal cells [27]. *LY6E* is a glycosylphosphatidylinositol-anchored stem cell antigen that belongs to the Ly-6 family and is involved in the self-renewal of erythroid progenitor cells [28], [29]. *SAR1A* encodes a guanosine triphosphatase which controls the assembly and fission of vesicular coat protein complex II on cellular membranes [30]. *EIF2S3* encodes a translation initiation factor that participates in the selection of the start codon. Phosphorylation of the α subunit of EIF2S3 at Ser-51 blocks the exchange of eIF2-GDP to eIF2-GTP, thus reducing global translation initiation and subsequent protein synthesis [31]. *TRIM13* encodes endoplasmic reticulum E3 ubiquitin ligase involved in protein degradation [32].

Transcripts downregulated in blastomere- relative to the whole embryo-derived lines include *UBE3B*, that encodes a HECT-domain E3 ubiquitin ligase which targets proteins for degradation [33]; *QK1* which product is RNA binding protein important for normal development in vertebrates and regulates protein translation, RNA splicing, export from the nucleus, and stability [34], [35]; *PASK* that encodes a PAS domain-containing protein kinase involved in regulation of many intracellular signaling pathways in response to both extrinsic and intrinsic stimuli [36] and *RAB3B* that encodes one of the Ras-associated GTP-binding protein 3 family members that regulates secretory vesicle transport between the Golgi apparatus and the plasma membrane [37].

**Figure 6:**
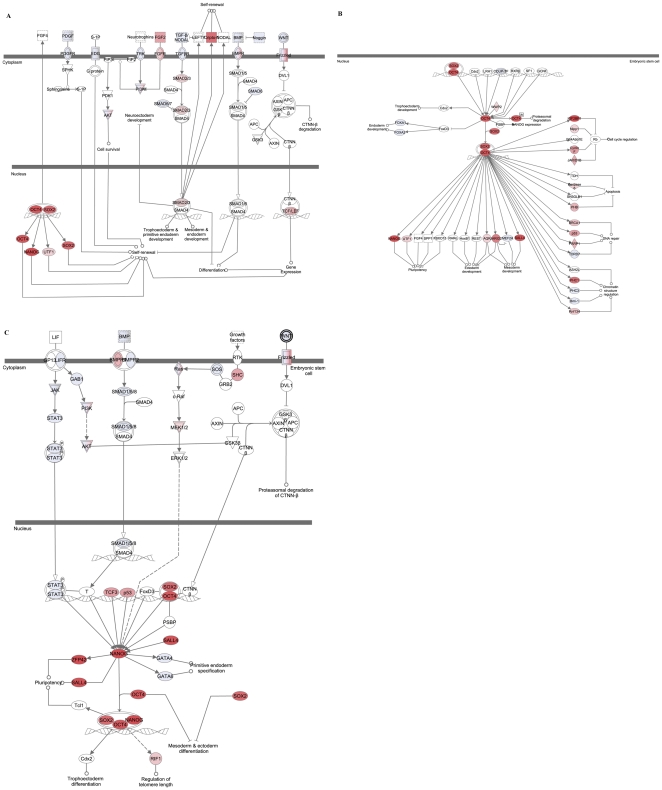
Key stemness pathways in cleavage stage blastomere-derived and blastocyst stage ICM-derived lines. Ingenuity Pathways Analysis showing that the key stemness pathways regulated by *OCT4* and *NANOG* are active in both blastomere-derived (A, C) and ICM-derived (B) hESC lines when compared to reference RNA.

The master regulators of human ESC stemness pathways are transcription factors *OCT4, SOX2* and *NANOG* [38]. They are critically involved in establishment and maintenance of pluripotent state in ESC and thus, closely associated with their stem cell identity and function. To identify whether key stemness pathways regulated by *OCT4, SOX2* and *NANOG* are active in both blastomere- and whole embryo-derived hESC lines we compared expression of genes involved in these pathways to reference RNA and, performed the pathway analysis using Ingenuity Pathways Analysis platform. The analysis revealed that a number of genes within these pathways such as OCT4, NANOG, SOX2 and SALL4 were overexpressed in both blastomere- and whole embryo-derived lines suggesting that hESC derived from two developmentally different stages of human preimplantation embryos, cleavage and blastocyst, maintain equivalent pluripotent states (Fig. 6).

## Discussion

In this study we have investigated whether differentiation status of source cells within preimplantation embryo affects phenotype and transcriptional profile of derived hESC lines. One of potential hurdles in performing such an analysis is the presence of differences between hESC cell lines that are not related to the developmental stage of the cells of origin. These differences may arise during cell line derivation and expansion as a result of *in vitro* selection of specific phenotypic and molecular traits/characteristics (“culture effect”). Another potential contributing factor to hESC line heterogeneity is diverse genetic makeup of the embryos due to outbred nature of human embryos. Consequently, despite the expression of a common pluripotent phenotype, hESC lines derived from single stage embryos, namely the ICM of the blastocyst, have been reported to show significant variations in both their molecular profiles and differentiation capacity [39], [40], [41], [42]. To minimize the effect of these factors on our investigation, we used for this study multiple hESC lines with different genetic backgrounds that were derived and cultured in our laboratory under virtually identical, well controlled, conditions. Indeed, our results suggest that once these culture-induced factors are eliminated, hESC derived from different stages of embryo development exhibit very little difference in their gene expression profiles and maintain similar pluripotent phenotype. One explanation for this finding is that although the source cells from which hESC are derived are clearly at different developmental stages, the process of hESC derivation selects for cells that assume certain phenotypic and molecular state required for the survival and self-renewal of pluripotent cells in culture.

One of the unique aspects of our analysis is that unlike the cell lines generally used for gene expression studies that were often derived and grown under heterogeneous culturing conditions and have typically undergone extensive passaging and selection in culture, lines used in this analysis were derived and grown under identical defined conditions for limited number of passages, thus minimizing culture-induced changes.

While overall transcriptional profile did not appear to be significantly altered between blastomere and ICM-derived hESC, we did detect a small number of genes with significantly different expression between two groups. Out of these, only *CLC*, *ZNF558* and *LGALS14* genes were down-regulated more than 4 fold in blastomere derived hESC lines. Interestingly, two of the genes encode proteins from galectin (galaptin/S-lectin) family characterized by presence of carbohydrate recognition domains and β-galactosidase binding activity. *CLC* whose expression is downregulated 20 fold, encodes a lysophospholipase, an enzyme that hydrolyzes lysophosphatidylcholine to glycerophosphocholine and a free fatty acid and thus, contributes to the regulation of cell membrane function. *LGALS14* gene is predominantly expressed in the placenta, an extraembryonic tissue that develops after blastocyst implantation. The significance of their low abundance in blastomere lines remains to be elucidated.

Our analysis does not preclude the possibility that there are additional subtle differences in molecular and phenotypic profiles between cleavage stage blastomere-derived and blastocyst stage ICM-derived hESC lines. For example, epigenetic changes including differences in DNA methylation patterns and X chromosome inactivation have been shown to be affected by different hESC states [43]. In addition, while we have not observed differences in the ability of blastomere-derived and ICM-derived hESC lines during spontaneous differentiation *in vitro* and *in vivo* to give rise to cells from all three germ layers, efficiency of differentiation and differentiation propensity towards specific cell lineages have not been evaluated and are subject of current investigation in our laboratory.

Nevertheless, overall our data strongly indicate that blastomere-derived lines exhibit all the key characteristics of hESC derived from ICM, including presence of pluripotency-associated pathways, ability to differentiate into cells from all three germ layers, self-renewal and hESC-specific transcriptional profile. This suggests that they represent a valid, embryo friendly, alternative to whole embryo derived lines for any future applications such as cell therapy and drug discovery.

## Supporting Information

Table S1 **RT-PCR primers and conditions.** FW and RV primer represent forward and reverse primer, respectively.
(DOC)
