# Supplementary material for: Human Embryonic Stem Cells Derived from Embryos at Different Stages of Development Share Similar Transcription Profiles
Source: PLoS One. 2011 Oct 21;6(10):e26570. doi: 10.1371/journal.pone.0026570 (PMC3198782; doi:10.1371/journal.pone.0026570)
Supplement: Table S1 — RT-PCR primers and conditions. FW and RV primer represent forward and reverse primer, respectively. (DOC) [file pone.0026570.s001.doc]

| **Gene** | **FW primer** | **RV primer** | **No. of PCR cycles** | **Anealing T** | **Amplicon size (bp)** |
| --- | --- | --- | --- | --- | --- |
| ***AFP*** | 5'-TgA AgA ggg AAg ACA TAA CTg-3' | 5'-AgC AgC CCA AAg AAg AAT-3' | 40 | 53 | 199 |
| ***TΥBB3*** | 5'-CTC Agg ggC CTT Tgg ACA TC-3' | 5'-CAg GCA gTC gCA gTT TTC AC-3' | 40 | 53 | 160 |
| ***T*** | 5'-ACT ggA TgA Agg CTC CCg TCT CCT T-3' | 5'-CCA Agg CTg gAC CAA TTg TCA Tgg g-3' | 40 | 57 | 568 |
| ***GATA4*** | 5'-CTg gCC TgT CAT CTC ACT ACg-3' | 5'-ggT CCg TgC Agg AAT TTg Agg-3' | 40 | 60 | 263 |
| ***NES*** | 5'-CAg CTg gCg CAC CTC AAg ATG-3' | 5'-Agg gAA gTT ggg CTC Agg ACT gg-3' | 40 | 60 | 208 |
| ***PAX6*** | 5'-AgA gAA gAC Agg CCA gCA AC-3' | 5'-CTT gAA CTg gAA CTg ACA CAC C-3' | 40 | 53 | 399 |
| ***ACTA2*** | 5'-CgT gTT gCC CCT gAA gAg CAT-3' | 5'-ACC gCC Tgg ATA gCC ACA TAC A-3' | 40 | 53 | 134 |
| ***SOX17*** | 5'-AgA ATC CAg ACC TgC ACA-3' | 5'-gCC ggT ACT TgT AgT Tgg-3' | 40 | 53 | 150 |
| ***WT1*** | 5'-gAT gAA CTT Agg AgC CAC CTT AAA-3' | 5'-TAT gTC TCC TTT ggT gTC TTT TgA-3' | 40 | 53 | 413 |
